# Factors promoting hunting groups’ sustainable harvest of moose in a co-management system

**DOI:** 10.1038/s41598-023-48348-2

**Published:** 2023-11-29

**Authors:** Laura S. Tuominen, Mikael Wikström, Heikki Helanterä, Patrik Karell, Jyrki Pusenius, Lauri Rapeli, Leena Ruha, Timo Vuorisalo, Jon E. Brommer

**Affiliations:** 1 University of Turku, Turku, Finland; 2 Finnish Wildlife Agency, Helsinki, Finland; 3 University of Oulu, Oulu, Finland; 4 Lund University, Lund, Sweden; 5 Natural Resources Institute Finland, Joensuu, Finland; 6 The Social Science Research Institute, Åbo Akademi University, Turku, Finland; 7 Natural Resources Institute Finland, Oulu, Finland

**Keywords:** Sustainability, Environmental economics

## Abstract

Collaboration between and within management levels and involvement of local communities (co-management) increases sustainable management of natural resources. In Finland, moose (*Alces alces*) are harvested by hunting groups within a co-management system, providing meat and social benefits. We computed the 14-year change in moose harvest (2007–2020) for 4320 hunting groups. Moose harvest declined on average 1.1% per year, but with substantial variation in moose harvest changes between the hunting groups. We extracted information describing the collaboration between the hunting groups, their democratic status as well as leader dynamics, and the year of establishment. A hunting group’s moose harvest was more stable (i.e. declined less) when the hunting group was (1) established a longer time ago; (2), had more changes in leadership over time, but did not depend on collaboration with other local hunting groups (in terms of jointly holding moose hunting licenses), whether the hunting group was a registered society (presumed to be more democratic than a non-registered one) or had consecutive leaders that shared a surname (presumed to be related). We conclude that encouraging resource users’ early establishment in groups and groups’ long-term persistence and promoting democratic leadership roles improves stable benefits from a natural resource in a co-management system.

## Introduction

Managing natural resources sustainably is an enduring challenge to human society. In particular, shared natural resources, the so-called commons, are vulnerable to over-exploitation when the selfish interest of resource users overrides cooperative sustainable use^1,2^. Sustainable management of shared natural resources often extends over several levels, involving—at the highest level—governance rules imposed by a state or country to—at the lowest level—the (groups of) individuals physically using the natural resource^3,4^. In addition, a natural resource is influenced by natural processes determining e.g. the rate at which it is renewed^5^. In general, the processes at these various levels are intertwined and the question of how to govern a natural resource sustainably requires an integrated study approach^6–8^. One such integrated approach is the Social-Ecological System framework^9^ which recognizes that natural resource management involves governance systems, resource systems, resource units, and resource users (actors)^10^. The rules, laws, regulations, and their enforcement are categorized under governance. Specific ecological properties of the resource systems and the resource units influence the probability of reaching sustainable resource management^2^. For example, mobile resource units such as wildlife, are challenging to manage as the resource boundaries cannot be defined well and their monitoring is difficult^11^. Lastly, attributes of resource users are of profound importance for the sustainability of the system as a whole^2^. For example, the socioeconomic status of resource users, their economic dependency on the resource, face-to-face communication and social capital between them often plays a role in reaching sustainable resource use^12–15^.

Adaptive co-management systems are offered as one solution to complex environmental problems, including the management of commons^13,16–18^. Collaboration between and within levels of management and involvement of local communities in decision-making can improve socially and ecologically sustainable resource use^3,19^. However, differences in local social and ecological conditions alter the outcomes of co-management^20^, and benefits to local communities or the environment vary^16,21^. In fisheries, for example, high social capital among the resource users and engagement of all stakeholders with a diversity of interests and socio-economic attributes has led to positive co-management outcomes^20,22^. However, unresolved conflicts or hidden power asymmetries, e.g. when the most powerful or wealthy resource users heavily influence the management decisions, can cause co-management to fail in its objectives^17,22^. There is evidence that adaptive co-management is suitable for wildlife management, where the objective is to both provide harvest to all resource users and manage the resource sustainably^13,23^. However, there is also evidence that co-management of wildlife requires more context-dependent solutions^24^. In particular, a deeper understanding of local social-ecological dynamics increases the probability of designing management matching these local ecological and social conditions and avoiding poorly working panacea solutions^2,25–27^.

**Figure 1:**
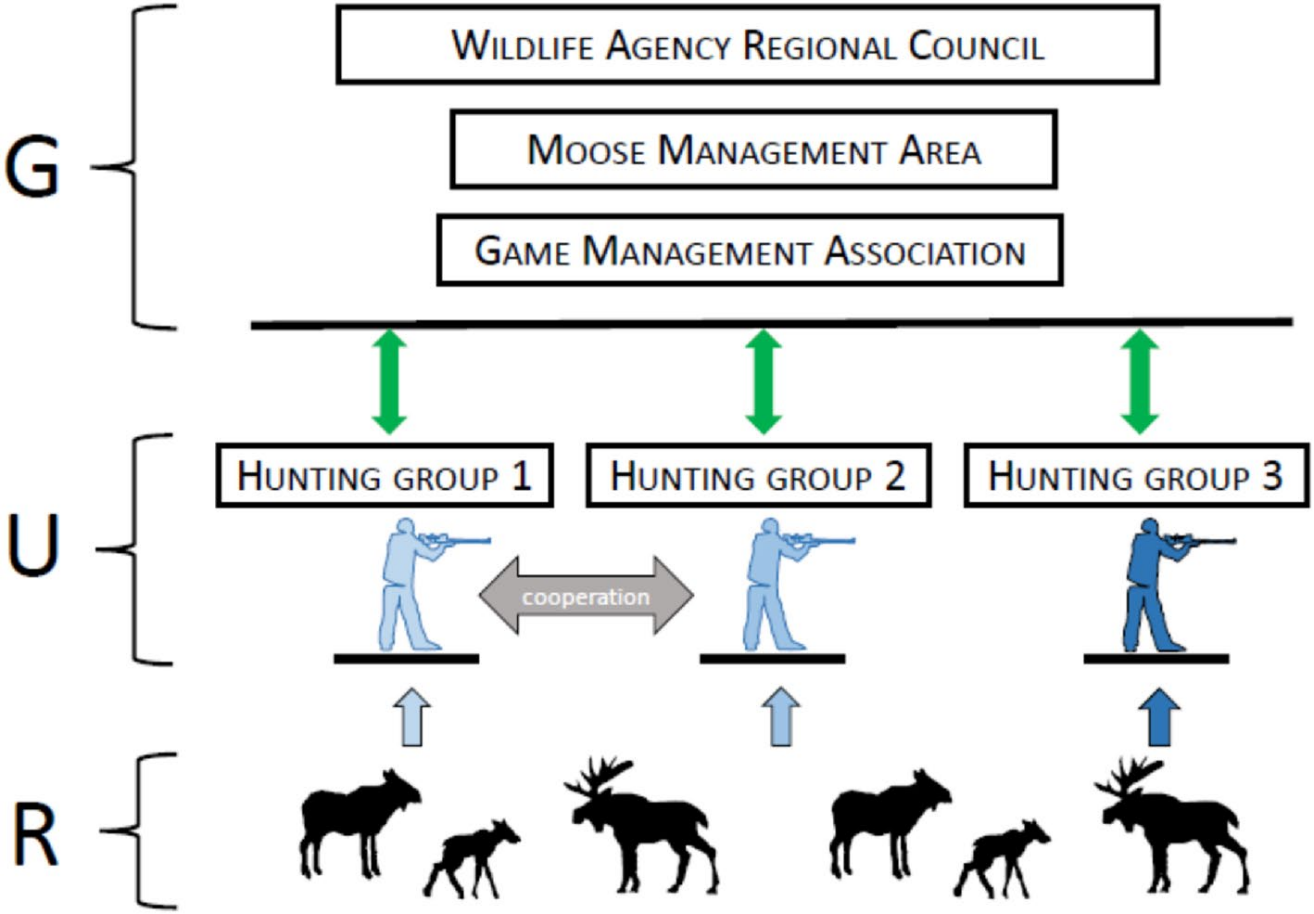
Schematic overview of the moose co-management system in Finland. The management concerns three levels; (G) governance, (U) resource users (hunting groups) and (R) wildlife resource (moose). Harvest targets are planned over 3-year period by regional wildlife councils (n = 15), which set goals that are specified as annual moose hunting licenses by the various moose management areas (n = 59) under them, together with the various game management associations (n = 282) belonging to a moose management area. Hunting groups are represented on all governance levels. Moose hunting licenses are applied for annually by hunting groups and rewarded by the governance level (green double arrows). Although based on licenses, the resource (moose) is harvested (one-sided blue arrows) by the hunting groups and these are free to not use all their licenses and to adjust their harvest of calves vs. adult moose. Hunting groups can also cooperate and share moose hunting licenses (grey double-sided arrow). Hunting groups differ from each other in characteristics (indicated by different blue coloring) that may affect their long-term (14-year) moose harvest trend.

Here we study moose, which is an integral part of boreal forest ecosystems^28^. However, it is also the economically most important wildlife species in Finland: moose meat is a valued natural resource, but at the same time, moose cause extensive damage to forestry and are involved in lethal traffic accidents^29,30^. Hence, various stakeholders (hunters, land-owners, and traffic safety authorities) often have conflicting target moose population numbers^31^. Moose numbers are regulated by hunting licenses, which are applied for and used by hunting groups but decided by a multi-level nationwide governance system (Fig. 1). Goals for moose density, sex, and age distribution are set every three years in 15 regional wildlife councils, further divided into 59 moose management areas, which plan the area’s harvest for one year at a time and are further divided into 282 game management associations (Fig. 1). The game management associations are in charge of the practical side of organizing local hunting and hunters. Therefore, the key to the Finnish moose governance system is adaptive co-management^32,33^: the explicit recognition of spatially divergent (i.e. regional and local) management. Regardless of the collaboration between the levels, the Finnish moose management system can still be largely characterized as top-down controlled through the strict license policy.

Here, we focus on the level of the resource users (i.e. hunting groups) in the moose co-management system. We ask whether characteristics of the local resource users can influence the long-term trend in the resources obtained (i.e. moose harvested) by these users (Fig. 1). To this end, we compute the geometric mean change in moose harvest over 14 years (2007–2020) for over 4000 moose hunting groups in Finland. Inspired by the Social-Ecological Systems framework^9,10^ we explore the influence of local hunting group dynamics on the long-term harvest. We study whether democratic decision-making (in terms of the hunting group being a registered society or not) and between-group collaboration (in terms of the hunting group sharing moose hunting licenses with other hunting groups or not) are associated with the hunting group’s long-term change in moose harvest. We use the Finnish public information act to obtain the names of the leaders of approximately 3400 registered hunting societies during the last 35 years, and use this information to study whether the frequency of leadership changes or the relatedness between the leaders is associated with harvest trends.

Our objective is to explore whether, within a co-management system of a wildlife natural resource, properties of groups of local resource users are associated with differential trends in harvest they are able to obtain. Although hunting licenses regulate moose harvest, moose hunting groups have flexibility in using these licenses; they can apply for licenses as they see appropriate, they are free to use their moose hunting licenses restrictively and thus harvest less than what they are legally allowed to, and hunting groups can further opt to use a license for one adult moose to harvest two calves. Hunting groups therefore have some power in regulating local moose population demography including the density of the moose population in their region and the population’s age and sex distribution^16^. Importantly, these demographic parameters play a key role in harvest dynamics, where primarily harvesting young individuals and striving to maintain a balanced sex ratio of a sufficient number of reproductive adults maximizes yield^34^. Hunting groups are therefore not necessarily equally capable to maintain a stable moose harvest locally. A stable moose harvest offers a reliable source of moose meat and recreational and social benefits for the hunting group and its members^30^. Therefore, for the groups of resource users, which is the level of focus in the study, the objective and socially sustainable harvest is a stable harvest rate over the years. Enjoying a stable harvest trend ensures social sustainability of the activity (hunting) by the local resource users and thereby assures the co-management system as a whole is sustainable. Although local-level processes are known to be important for natural resource systems in general, it has, to our knowledge, not been studied whether local-level management processes are associated with long-term harvest trends of wildlife^8,9,35,36^.

## Materials and methods

### Hunting group classification

Many hunting groups are registered societies at the Finnish Patent and Register Office. From here on, we call these hunting groups registered hunting societies. A registered hunting society is to follow the Associations Act (503/1989 and its amendments). In brief, a registered society must have at least two meetings per year for all its members, and it must have a steering board (chair, possibly vice chair, possibly secretary, and other board members) chosen by its members. Other, relatively rare, formal organizations that form a hunting group include company and group-owned forests. Moose hunting licenses can also be applied for by a hunting group that has no formal organization. We term this latter category a non-registered hunting group. A non-registered hunting group typically is a coalition of individuals who together have hunting rights on a sufficient amount of land (1000 ha or more) to qualify for moose hunting licenses. A non-registered hunting group is not required by law to follow any democratic decision-making process.

The Wildlife Agency does not require applicants for moose hunting licenses to state whether they represent a registered hunting society or a non-registered hunting group. However, the applicant needs to provide a name for the license holder. The applicant is assigned a unique hunting group ID, which remains the same over the years. We here used the names provided for the annual moose hunting licenses to classify each hunting group ID. We considered the hunting group as a registered hunting society if its name was found in the public registry of registered societies maintained by the Finnish Patent and Register Office (URL: https://yhdistysrekisteri.prh.fi/?userLang=en), otherwise it was considered to be a non-registered hunting group. Some hunting groups also registered themselves during the study period and were categorized as registered hunting societies for the whole study period.

### Classification of management units

Each hunting group belongs to one game management association. During the study period, some game management associations were combined in an administrational reform. For consistency, we categorized each hunting group to belong to the game management association they were part of in 2020. In addition, Finland was in 2015 divided into 59 moose management areas, which do not always follow the game management association borders. For consistency across the study period, we categorized each game management association to belong to one moose management area. In case a game management association belonged to two moose management areas, we considered that game management association to belong to the moose management area that covered most of its area. The moose management areas and game management associations fall under 15 regional Wildlife Agency offices.

### Change in moose harvest and moose population size

Hunting groups that have a moose hunting license are required to report how many moose adults and calves were harvested each hunting season. We here tally “adult equivalents”, where harvesting one calf was counted as ½ adult. The rationale for doing so was based firstly on the fact that hunting groups can opt to change two licenses for calves to a license for one adult. Furthermore, a calf has a dressed weight (weight after slaughter) of around half (80 kg) of an adult [170 kg (female) to 180 kg (male)].

We computed the geometric mean annual change in moose harvest during 2007–2020 for hunting group *g* with non-zero “adult equivalent” moose harvest *H* starting in year *f* and ending in year *l* as $\lambda_{g} = \left( {H_{g,l} /H_{g,f} } \right)^{{\frac{1}{1 + l - f}}} ,$ where the denominator in the exponent denotes the number of years between first and last non-zero harvest. This formulation ignores information on harvest between the first and last year, but is mathematically equivalent to computing the geometric metric using all annual harvests (Electronic Supplement). Although years without harvest are hence ignored in this approach, they were relatively rare (see Results). Stable long-term harvest was achieved when *λ*_*g*_ is one, whereas a value of *λ*_*g*_ below unity implies harvest was, on average, declining. The long-term change in harvest *λ*_*g*_ was computed for hunting groups harvesting moose in more than 10 years.

### Proportion of moose hunting licenses used and use of shared moose hunting licenses

From 2016 onwards, the Wildlife Agency assigned an additional unique moose hunting license number to each license application. We computed for these years for each moose hunting license number the proportion of moose harvested under that license. In case the harvested moose is not suited for consumption (e.g. due to disease), hunting groups can get permission to harvest a replacement under the same license. Thus, the number of harvested moose may exceed the licensed number and the proportion of licenses used hence may exceed 1.

Hunting groups can jointly apply for moose hunting licenses. When moose hunting licenses are shared, the division of the licensed animals is left to the hunting groups that share the licenses, requiring some level of collaboration. Hunting groups are, however, required to individually report moose harvest also under a shared moose hunting license. Hunting groups were considered to share licenses if they harvested moose during one or more years (out of the five years considered) under the same moose hunting license number. Typically, if a hunting group shared moose hunting licenses they did so during all five years considered.

### Variables describing a registered hunting group

Information about the date a society is registered can be obtained from the public registry of societies (link provided above). Societies are furthermore to report to the Finnish Patent and Register Office the rules and/or the names of the society members that have signature rights, which is public information available upon request. We requested from the Finnish Patent and Register Office for all registered hunting societies included in our dataset all records of signature rights, including the names of the persons. Signature rights in a society are typically assigned to leading members of the steering board of the society: the chair, and/or vice-chair, and/or secretary of the board. The Finnish Patent and Register Office provided records for most registered hunting societies in the form of a PDF, although it was unable to provide records for 99 registered hunting societies. Signature rights are reported to the Finnish Patent and Register Office starting in the 1980s. To measure the stability of the steering board of a registered hunting society, we scored how many times the names of leading board members (chair, vice-chair, secretary) changed since 1985. Typically, the names provided consisted of first, second, and surname and these names were typically male names. As a measure of board members potentially being relatives, we further scored whether two or more board members who did not share first and second names, shared a surname or not.

### Statistical analyses

The mean change in harvest was analyzed using a linear mixed model. The fixed effects were whether the hunting group was a registered hunting society or a non-registered hunting group and whether the hunting group had shared a license or not shared a license. In a second model that only considered registered hunting societies also the year the society was established, the number of changes in the leading board members and whether board members shared a surname or not were included as fixed effects. In both models, random effects were Wildlife Agency region, moose management area, and game management association. These random effects corrected for the non-independence of hunting groups across different spatial units. The proportion of variance explained by each random effect was computed as the ratio of each variance component over the sum of variance components. All linear mixed models were solved using Restricted Maximum Likelihood (REML) implemented in AsReml in R^37^. Fixed effects were tested using Wald statistics, reported as a chi-square test. Random effects were tested using a Z ratio of the estimate and its standard error. Residuals of the linear mixed model were verified to follow an approximate normal distribution. Prediction intervals were created using the predicted means and standard error. The standard error of the proportion of variances was computed using the delta method.

## Results

### Trends in moose harvest on the level of hunting groups

There were 4 320 hunting groups with more than 10 years of moose harvest during 2007—2020. Out of these 4 320 hunting groups, 82% (3 537) were registered hunting societies, 0.5% (24) were hunting groups organized in another way (company or group-owned forest), 17% (742) were non-registered hunting groups, and for 0.4% (17) their organization could not be resolved unequivocally. We focus further analyses on the 4 279 hunting groups that are either a registered society or a non-registered hunting group.

**Figure 2:**
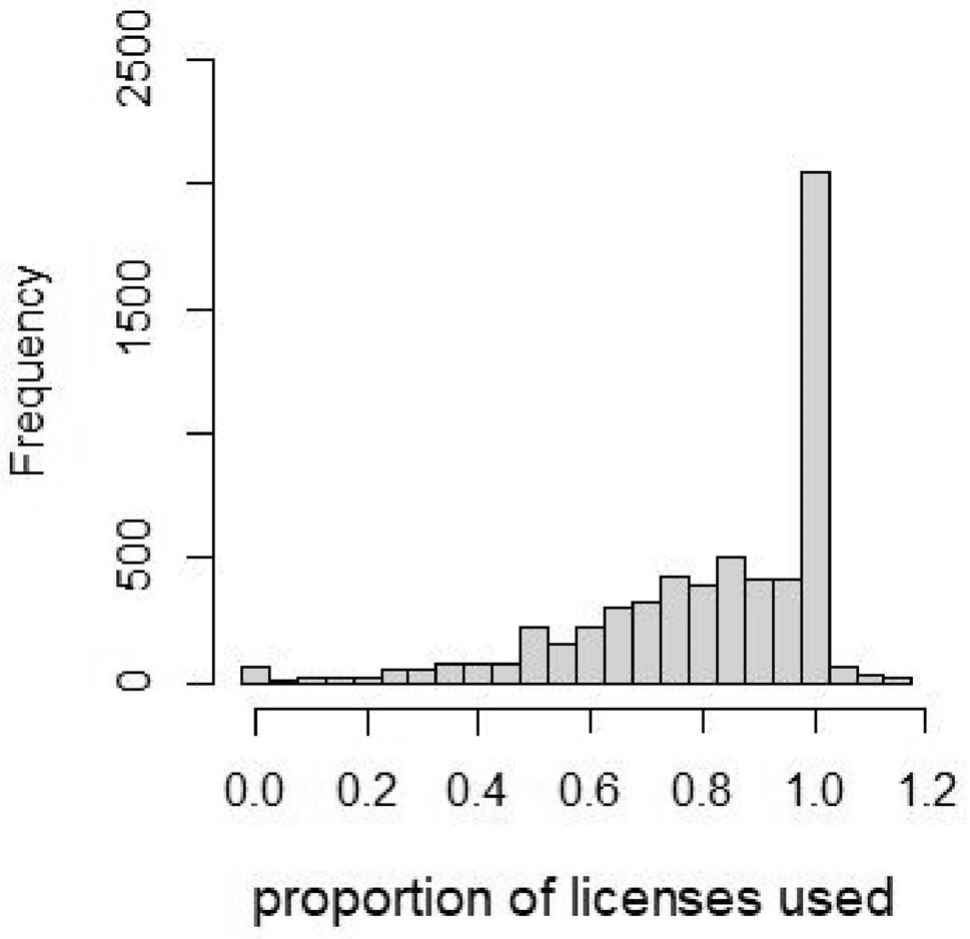
Frequency distribution of the proportion of moose hunting licenses that resulted in harvested moose during the years 2016–2020. During these years a total of 6 054 hunting licenses were provided to the hunting groups included in this study. The (few) cases where hunting groups harvested more moose than they had moose hunting licenses (i.e. proportion greater than 1) are because they obtained special dispensation for doing so. For visual clarity, the plot omits 17 moose hunting licenses where the proportion harvested exceeded 1.2 because these were due to small number of licenses.

The majority (88%) of these hunting groups harvested moose during the entire 14-year study period (14: *n* = 3 764; 13: *n* = 274; 12: *n* = 131; 11: *n* = 110). For the 515 hunting groups that did not harvest moose in all 14 years, the absence of harvest in one or more years was for most of these hunting groups (89%, 457/515) due to not obtaining (or even applying for) a moose hunting license rather than not harvesting moose despite having moose hunting licenses. While these hunting groups typically harvested the maximal number of moose they had moose hunting licenses for, it was not uncommon to harvest fewer moose than the licensed number (Fig. 2); indeed, on average, these hunting group harvested 81% of the moose they had licenses for during 2016–2020.

**Figure 3:**
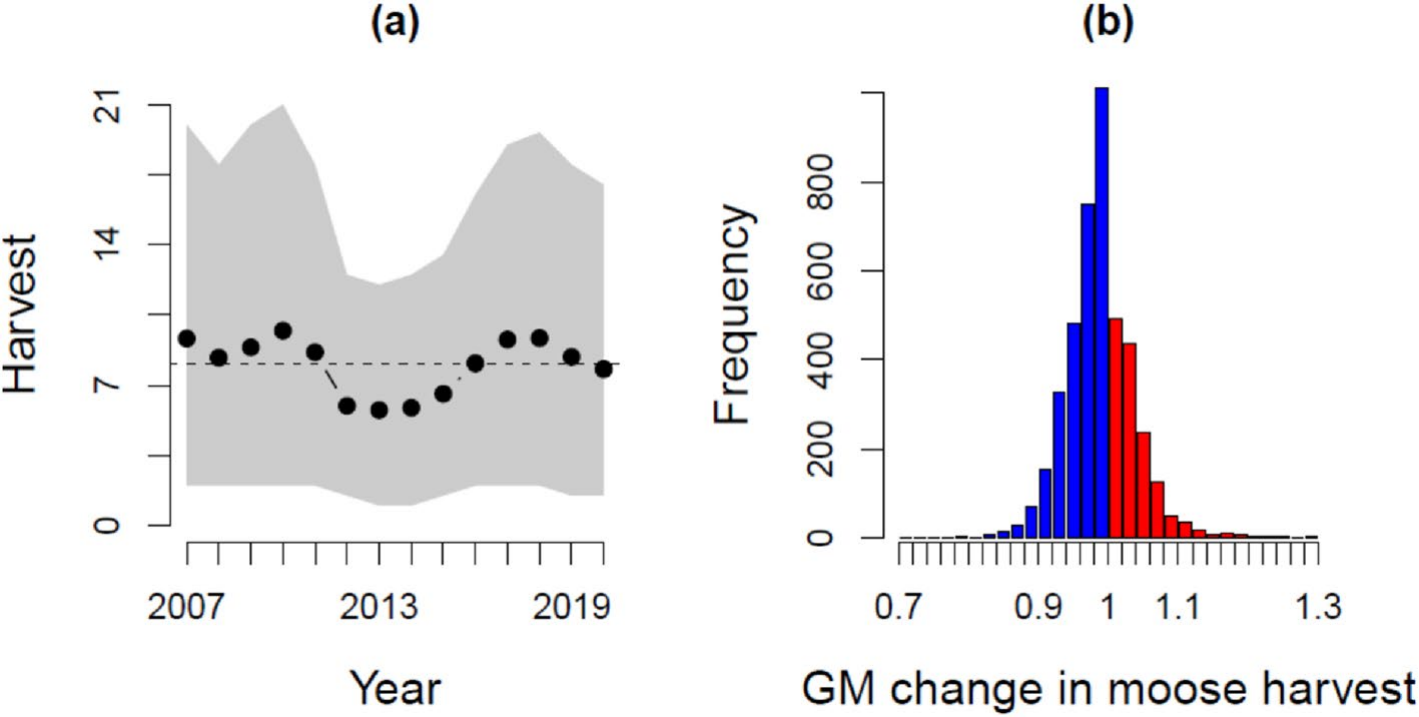
Plots of (**a**) the annual moose harvest per hunting group, and (**b**) long-term geometric mean proportional change in harvest for 4279 hunting groups during 2007–2020. In (**a**), the number of harvested moose are expressed as adult moose equivalents by counting each harvested calf as half an adult with plotted the mean annual harvest per hunting group (dots and line) with its 82.4% quantiles (grey area). In (**b**), the frequency distribution of the geometric mean (GM) change in moose harvest is plotted, where thin horizontal lines denote a frequency of zero. GM changes that constitute a decline in harvest are plotted in blue and those with an increase in red.

There was considerable variation in moose harvest between hunting groups across years (Fig. 3a). On average, a hunting group harvested 8.0 moose adult equivalents annually, totaling 31 939 adult moose equivalents per year, and a majority (88%, 447 142/510 687) of the total number of moose harvested in Finland during the study period, assuring these groups are representative of Finnish hunting groups. A hunting group’s moose harvest declined on average by approximately 1.1% annually (Fig. 3b; mean = 0.988, 95% quantiles 0.893:1.088). Most (87%; 3 703) of these hunting groups shared a moose hunting license with at least one other hunting group. However, harvest trends did not differ between non-registered hunting groups and registered hunting societies (${\chi }_{1}^{2}$ = 0.06, *P* = 0.80) or between groups that shared or never shared a moose hunting license (${\chi }_{1}^{2}$ = 0.15, *P* = 0.69).

### Properties of hunting societies affecting their long-term harvest

**Figure 4:**
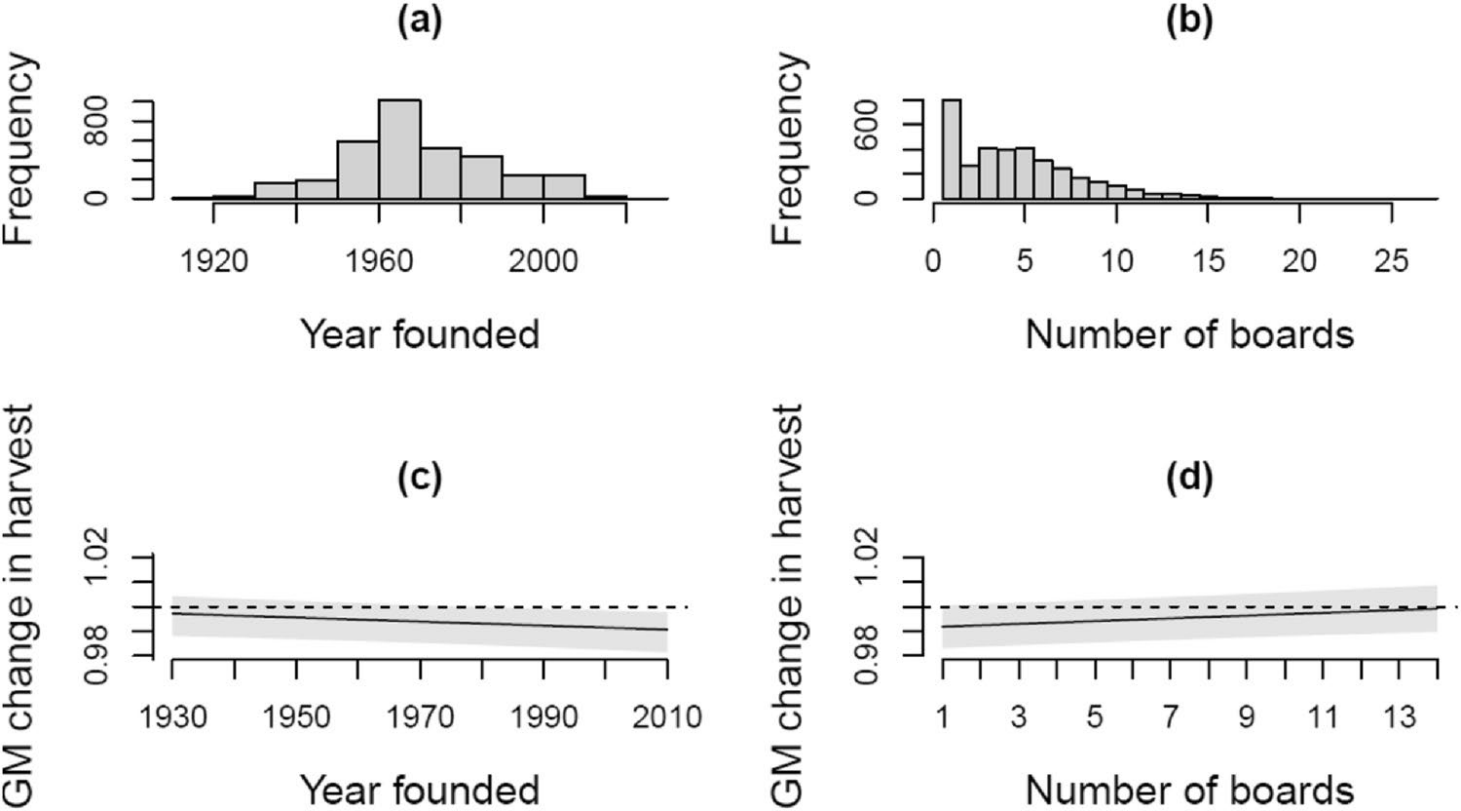
Frequency distributions (**a**, **b**) and prediction with prediction interval (**c**, **d**) for the fixed effect significantly affecting geometric mean change in moose harvest for 3434 registered hunting societies. In (**a**) and (**b**), the frequency distribution of the founding year and number of different boards, respectively, of the societies are plotted. In (**c**) and (**d**), model predicted geometric mean change in moose harvest and its 95% prediction interval (in grey) is plotted against the founding year and number of boards of the societies, respectively. Model details provided in Table 1.

There was information on signature rights available for 3 438 registered hunting societies. On average, these hunting societies were registered in 1970 (Fig. 4a), and had 4.7 different boards on average (median 4) since 1985 (Fig. 4b). In 52% (1 785) of registered hunting societies, board members shared a surname. When surnames were shared, on average 23% of all board members shared a surname with another board member. The moose harvest was more stable (geometric mean approaching one) in older hunting societies (Table 1; Fig. 4c), and in hunting societies where leading board members changed more often (Table 1; Fig. 4d). Whether board members shared a surname or not did not affect harvest trends (Table 1). In addition, differences across spatial blocks were important: wildlife agency region and game management association explained about 12% and 24%, respectively, of the variance in long-term changes in moose harvest. Differences in moose management areas explained about 7% (Table 1).

**Table 1:** Mixed model of the geometric mean proportional change in moose harvest of 3434 registered hunting societies during 2007–2020, belonging to 279 different game management associations in 58 different moose management areas operating under 15 Wildlife Agency Regions.

Fixed	Estimate	SE	Wald χ^2^	P
Intercept	0.994	0.00520		
Not shared a license	− 0.0007	0.0022	0.20	0.65
Year founded	** − 8.19e−05**	**4.69e−05**	**11.4**	** < 0.001**
Number of boards	**5.65e−04**	**2.56e−04**	**6.7**	**0.010**
Board members shared surnames	3.38e−4	1.57e−3	0.046	0.83

Random	Estimate (× 10^−4^)	SE (× 10^−4^)	Z	Prop
Wildlife Agency Region	2.99	1.47	2.03	**0.12**
Moose Management Area	1.79	0.90	1.99	**0.07**
Game Management Association	5.82	0.75	7.71	**0.24**
Residuals	13.72	0.35		**0.56**

Fixed and random effect estimates are provided with their standard error (SE), and test statistics. Fixed effects were tested with a Wald chi-square test and random effect variance components using the Z test. The fixed effect “Not shared a license” is the contrast to hunting groups that shared a moose hunting license during 2016–2020. For random effects, also the proportion of total variance explained for each random effect (prop) are provided. The fixed effect “Year founded” is standardized to have the mean year (1970) as zero. The fixed effect “Number of boards” notes the number of times the society was led by different boards (chair, vice-chair, secretary) as reported to the Finnish Patent and Register Board since 1985, and is relative to its median 4. The fixed effect “Board members shared surnames” denoted whether surnames were shared among leading board members each time they changed. Significant fixed effect and (for random effects) significant proportions of variance explained are printed in bold.

## Discussion

Social-Ecological Systems framework^2,9^ studies highlight that the lowest level of decision-making impacts management. We here explore whether differences between hunting groups, the lowest level operating within a wildlife resource co-management system, are associated with differential management outcomes. Hunting groups play an important role in the management system, as they implement the national and regional moose management plans^14^. Based on 14 years of moose harvest data, we find the moose harvest to, on average, decline by about 1% per year. That is, the moose harvest in 2020 declined, on average, to 87% (0.99^14^) of the initial harvest in 2007. Nevertheless, there is substantial variation between hunting groups in these harvest trends. This variation allows the investigation of the dynamics and outcomes of moose hunting on a local (hunting group) scale^20^. We find that hunting groups that are older and more often change their leaders enjoy a more stable (less declining) harvest trend during the study period.

Wildlife, such as moose, is a valuable natural resource economically, socially, and ecologically^16^. The hunting groups studied here harvest each year on average more than 30 000 moose adults or adult equivalents (two calves), worth roughly 30 M€ (conservatively assuming 100 kg meat per adult/two calves valued at €10/kg). Nevertheless, moose—as other wildlife—are part of the commons^8^. Hence, over-exploitation (the tragedy of the commons^1^) is always a threat and has been common historically^38^. For moose, as for other wildlife resources in general, therefore, strict management rules, operationalized by permits and licenses, are in place to regulate hunting to avoid overexploitation and to assure sustainable harvest. The overall trend in moose harvest showed an annual decline during the 14 years studied, which is in line with the management objective during this time period to reduce moose density and thereby minimize traffic accidents and forest damage. Despite the management objective, a stable harvest where similar amounts of moose can be harvested also the next years is optimal for the hunting groups. Hunting groups can, because they implement the hunting, affect long-term trends. For example, hunting groups can opt to not harvest all moose for which they have a license, which we here show to be relatively common. By influencing the moose population demography through hunting decisions, hunting groups have the potential to influence harvest trends. We indeed observe a wide variance in harvest trends between hunting groups and find certain hunting-group-level characteristics to correlate with a more stable harvest. At the same time, this finding implies that a potential conflict may arise in a co-management system. This is because objectives at different levels of the management system cannot differ considerably without risking the long-term sustainability of the system as a whole. In the long run, therefore, objectives should be aligned at the various levels of the co-management system.

We find that older registered hunting societies enjoy a more stable harvest trend compared to newer ones. This finding mirrors previous results in the management of fisheries, community forests, and overall commons, where experiences in collective action and knowledge about the area’s social and ecological context improve management and lead to better outcomes^2,17,39^. Importantly, in the more than 100 years since the first registered moose hunting societies were founded, the Finnish moose population has undergone dramatic changes. In the 1930s, only a few thousand moose were left in Finland. A partial recovery was again followed by a drastic decline resulting in a ban on moose hunting instated in most of Finland in 1969–1970^40^. These challenges may have created a situation where only well-functioning hunting societies remain with hunting societies functioning less well halting their activities, although it should be noted that most hunting societies also hunt other wildlife than moose. A further non-mutually exclusive aspect is that these older (remaining) societies may be located in areas that are ecologically favorable for moose and because of that were able to continue harvest at some level despite drastic fluctuations in moose abundance.

We further find that registered hunting societies with more frequent leadership changes enjoy a more stable moose harvest trend. This finding contrasts with the notion that a lasting trust in leaders creates stability in the group with higher incentives for cooperation leading to better management^23,41,42^. In general, seemingly democratic co-managed resource systems may have risks of power abuse or power asymmetries^43^. The benefits, i.e. harvest received by the individual hunters, can be affected by the power division^3,17^ and power hoarding may therefore reduce the functioning of the group. We see two non-exclusive alternative mechanisms for why frequent changes in leading board members seem to be positive for the groups. Firstly, frequent leadership changes may signal a higher readiness to allow new leaders to enter, implying well-functioning democratic arrangements (no power hoarding) and high within-group social capital^14,44^. Societies can, for example, have agreed on statutory rules that specify a maximum period of time steering board members can be in power. Secondly, more changes in leadership may be due to there being more group members interested in taking leadership roles, and hence in sharing responsibilities, implying well-working group dynamics and high member commitment to the group^45^. Further evidence that a dynamic social structure plays an important part in well-functioning Finnish hunting groups stems from a survey study of these hunting groups which found that regular addition of new members into the group as well as commitment of members to the group are beneficial (Tuominen et al. in prep). Overall, therefore, we believe a higher leader turnover signals an active and democratic hunting society with engaged members providing an ability to adapt to changes in the ecological and social environment correlating with a more sustainable resource management^45–47^. However, we acknowledge that there can be some underlying dynamics in the hunting groups that are associated with frequent leadership turnover leading to stable harvest over the years. The dynamics related to leadership and its change in the hunting groups are not well known and a deeper understanding of them could provide insight into the issue.

Registered societies are—by law—required to have two meetings per year for all members where, among others, the functioning of the steering board and society are evaluated by its members. Hunting groups that are registered hunting societies are therefore expected to adhere to democratic decision rules. We find that these registered hunting societies do not differ in their long-term change in moose harvest compared to non-registered hunting groups that do not need to adhere to democratic decision-making rules. Our findings, therefore, suggest that having a democratic decision-making mechanism does not influence the long-term harvest of resources. This finding is in contrast to studies of fisheries management, where non-democratic groups are more effective or resilient to changes^43^. However, in a survey of Finnish hunting groups, Tuominen et al. (in prep.) found that members of registered hunting societies often reported being left out in the decision-making: A registered hunting society may hence be less democratic than intended by regulations. Furthermore, non-registered hunting groups may in practice adhere to a similar level of democratic decision-making as registered hunting societies^43^. We find that the collaboration between the hunting groups, against expectations^48^, does not influence the long-term stability of moose harvest. In contract, a survey study of Finnish moose hunters found that collaboration between hunting groups influenced positively the group management (Tuominen et al. subm). It therefore seem that collaboration through license-sharing, allowing information sharing, coordination of strategies, and a wider view of resource state between neighboring groups, does not influence long-term harvest dynamics^14,19,49^.

## Conclusion

Previous research on moose management^24,31^ has underlined that management decisions made on large spatial scales, and regional ecological conditions are important aspects in wildlife resource management. However, co-management not only aims to achieve sustainable ecological outcomes or benefits for the community at large. The objective and—arguably—the foundation of co-management is to provide a stable yield to the lowest level, the resource users. At the resource user level, a more stable yield is socially sustainable ensuring the sought benefits, such as meat and hunting experiences. Social sustainability at the resource user level also increase the probability of a sustainable management system as a whole when resource users are satisfied and commit to the activity. We find that some characteristics of local hunting groups (the resource users^9^) make it more likely to reach a stable long-term moose harvest. In designing co-management strategies on a local scale, our findings produce two recommendations. First, the co-management system should support early establishment, formalization, and organization of the resource users into groups as well as groups` long-term persistence as we find that early established groups enjoy a more stable long-term harvest trend. Second, the co-management system should encourage democratic rules assuring regular turnover of leaders of the groups of resource users to ensure that power and responsibilities within the groups are shared, as we find leadership changes promote stable long-term harvest trends. Changes in leaders make it less likely that the decisions taken by the resource users’ groups will benefit solely the uncontested leaders but instead will benefit the group and the resource. Clearly, these solutions are context-dependent, and therefore the system needs to be flexible allowing adaptation over time and space^2,24,35^. Therefore, we suggest that the recommendations should not be forced but rather provided to the hunting groups and their effect monitored over time. An implication of our finding is that for the co-management system as a whole to be sustainable, attention should be paid that interests at the level of the resource user is in line with higher management levels. Indeed, our study brings forward that in co-management systems, a deeper understanding of local communities implementing resource management is valuable for improving the sustainable use of natural resources.

### Supplementary Information

Supplementary Information.

## Data Availability

The datasets used and/or analysed during the current study available from the corresponding author on reasonable request.
