# Supplementary material for: Factors promoting hunting groups’ sustainable harvest of moose in a co-management system
Source: Sci Rep. 2023 Nov 29;13:21076. doi: 10.1038/s41598-023-48348-2 (PMC10687258; doi:10.1038/s41598-023-48348-2)

Electronic supplement

In this supplement we elaborate on the computation of geometric mean proportional change used in our paper and illustrate the equivalence of the approach to a regression approach.
By taking the geometric mean of a series of proportional changes, all numbers between the first and last proportional change are cancelled out. Suppose, we have a series of *n* numbers; x_1, x_2, x_3, …, x_n, the geometric mean of the proportional changes of the series is then

$$\left({x_2}/{x_1} \cdot {x_3}/{x_2} \cdot \ldots \cdot {x_n}/{x_{n-1}}\right)^{1/n} = \left({x_n}/{x_1}\right)^{1/n}. \quad (1)$$

Alternatively when computing the geometric mean in logarithmic notation:

$$\exp\left({\ln\left({x_2}/{x_1}\right) + \ln\left({x_3}/{x_2}\right) + \cdots + \ln\left({x_n}/{x_{n-1}}\right)}/{n}\right) =$$
$$\exp\left({\ln(x_2) - \ln(x_1) + \ln(x_3) - \ln(x_2) + \cdots + \ln(x_n) - \ln(x_{n-1})}/{n}\right) = \exp\left({\ln(x_n) - \ln(x_1)}/{n}\right) =$$
$$\exp\left({\ln\left({x_n}/{x_1}\right)}/{n}\right) = \left({x_n}/{x_1}\right)^{1/n}. \quad (2)$$

The latter logarithmic formulation also clarifies the equivalence between the geometric mean proportional change and a linear regression computed for the logarithm of the *n* values against the index of the series (1, 2, …, *n*) . This is because the slope of this regression then infers the average proportional change in the logarithmic values over the series (i.e. (2) above), bar some differences due to using regression methodology to infer this average rather than direct computation. To substantiate this statement, we constructed a small simulation in R of 1000 time series randomly varying around a constant mean change. The script is below and produces amongst others a plot showing the good correspondence between the geometric mean as we compute it (based on the first and last harvest; log(gm.mean.lambda; plotted on Y) and a regression approach (reg.coefs; plotted on X). The correlation between these two is high (on average 0.952 based on 100 simulations) and the line drawn shows 1:1 correspondence. It seems the regression approach produces slightly larger estimate of mean proportional changes for values further away from the overall mean, but clearly the correspondence between the approaches is very high.

```
#R script
# trend in harvest
# geometric mean and regression
compute.cor<-function(
mean.lambda=1.0, #constant on average
sd.lambda=.12, #some variance around the average
n=10, #number of time steps
G=1000, #number of groups
draw=TRUE)  #logical, whether graphs are drawn or not
{
#start values
h=matrix(rnorm(n,100,10),G,n) #random start values
for (t in 2:T.steps) {
  lambda<-rnorm(G,mean.lambda,sd.lambda) #random "annual" lambdas for groups
  h[,t]<-h[,(t-1)]*lambda #compute harvest in next time step
}
# -- all harvest trends
if (draw) { #simple plots of the series over time
plot(h[1,],type='l',ylim=c(0,300))
sapply(2:G,function(x) lines(h[x,],type='l'))} #geometric means
#-- compute geometric mean using the approach in our paper
```

```
gm.lambda<-(h[,T.steps]/h[,1])^(1/T.steps)
# -- compute the regression coefficients of the log's
reg.coefs<-sapply(1:G,function(x) lm(log(h[x,])~I(1:T.steps))$coefficients[2])
# -- plot the GM computation against the regression coefficients
if (draw) {
  plot(exp(reg.coefs),gm.lambda)
  abline(0,1) #1:1 line
}
return(cor(reg.coefs,log(gm.lambda))) #correlation
}
compute.cor() #one run with plots
cors<-sapply(1:100,compute.cor,draw=F) #more runs, no plotting
mean(cors)
```

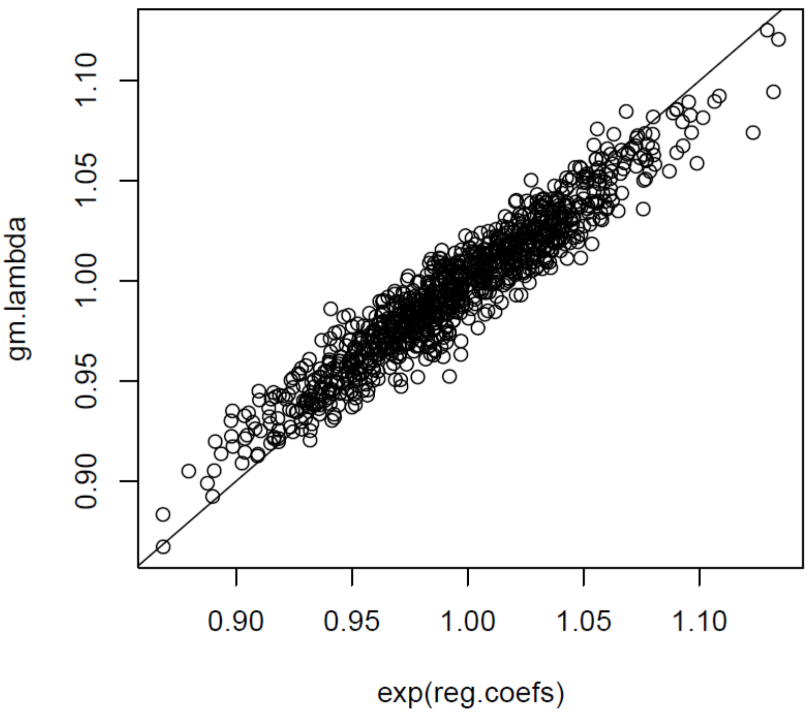

Supplement: Supplementary file 1 — Supplementary Information. [file 41598_2023_48348_MOESM1_ESM.pdf]
